# Defining the value of medical microbiology consultation

**DOI:** 10.1128/jcm.00359-24

**Published:** 2024-06-21

**Authors:** Kyle G. Rodino, Paul M. Luethy, April N. Abbott, Jeffrey M. Bender, Allison R. Eberly, Melissa Gitman, Amy Leber, Jennifer Dien Bard, on behalf of the Personnel Standards and Workforce Subcommittee, American Society for Microbiology

**Affiliations:** 1 Department of Pathology and Laboratory Medicine, Perelman School of Medicine, University of Pennsylvania, Philadelphia, Pennsylvania, USA; 2 Department of Pathology, University of Maryland School of Medicine, Baltimore, Maryland, USA; 3 Department of Laboratory Medicine, Deaconess Health System, Evansville, Indiana, USA; 4 Department of Pediatrics, Children’s Hospital of Los Angeles, University of Southern California, Los Angeles, California, USA; 5 Department of Pathology and Immunology, Washington University School of Medicine, St. Louis, Missouri, USA; 6 Department of Pathology, Molecular and Cell-Based Medicine, Icahn School of Medicine at Mount Sinai, New York, New York, USA; 7 Department of Laboratory Medicine, Nationwide Children's Hospital, The Ohio State University College of Medicine, Columbus, Ohio, USA; 8 Department of Pediatrics, Nationwide Children's Hospital, The Ohio State University College of Medicine, Columbus, Ohio, USA; 9 Department of Pathology and Laboratory Medicine, Children’s Hospital Los Angeles, Keck School of Medicine, University of Southern California, Los Angeles, California, USA; NorthShore University HealthSystem Department of Pathology and Laboratory Medicine, Evanston, Illinois, USA

**Keywords:** medical microbiologist, clinical microbiology, clinical consultation

## Abstract

Medical microbiologists, defined as doctoral-level laboratory directors with subspecialty training in medical microbiology, lead the clinical laboratory operations through activities such as clinical consultations, oversight of diagnostic testing menu, institutional leadership, education, and scholastic activities. However, unlike their clinical colleagues, medical microbiologists are largely unable to bill for clinical consultations performed within the hospital and, therefore, unable to generate relative value units or a similar quantifiable metric. As hospital budgets tighten and justification of staffing becomes a necessity, this may present a challenge to the medical microbiologist attempting to prove their value to the organization. To aid in providing tangible data, the Personnel Standards and Workforce subcommittee of the American Society for Microbiology conducted a multi-center study across seven medical centers to document clinical consultations and their impact. Consults were generated equally from internal (laboratory-based) and external (hospital-based) parties, with the majority directly impacting patient management. Near universal acceptance of the medical microbiologist’s recommendation highlights the worth derived from their expertise. External consults required more time commitment from the medical microbiologist than internal consults, although both presented ample opportunity for secondary value, including impact through stewardship, education, clinical guidance, and cost reduction. This study is a description of the content and impact of consultations that underscore the importance of the medical microbiologist as a key member of the healthcare team.

**IMPORTANCE:**

Medical microbiologists are invaluable to the clinical microbiology laboratory and the healthcare system as a whole. However, as medical microbiologists do not regularly generate relative value units, capturing and quantifying the value provided is challenging. As hospital budgets tighten, justification of staffing becomes a necessity. To aid in providing tangible data, the Personnel Standards and Workforce subcommittee of the American Society for Microbiology conducted a multi-center study across seven medical centers to document clinical consultations and their impact. To our knowledge, this is the first study to provide detailed evaluation of the consultative value provided by medical microbiologists.

## INTRODUCTION

Medical microbiologists are highly trained, doctoral-level (Ph.D., M.D., or D.O.) laboratory directors. The majority have completed fellowships in medical microbiology accredited by the subCommittee on Postdoctoral Educational Programs (CPEP) or the Accreditation Council for Graduate Medical Education and subsequent certification exams offered through the American Board of Medical Microbiology (ABMM), the American Board of Pathology (ABP), or the American Board of Bioanalysis (ABB), meeting the education and training requirements of High-complexity Laboratory Directors per the Clinical Laboratory Improvement Amendments. This extensive training in the subspecialty of medical microbiology prepares individuals to serve as essential members of the healthcare team (1).

The spectrum of roles the medical microbiologist is responsible for include, but are not limited to the following: serving as medical director for clinical consultation, oversight of, and long-term vision for, the diagnostic microbiology laboratory, regulatory and administrative leadership, education, and scholastic activities. Doctoral-level medical directors with training in subspecialities, such as medical microbiology, are often underappreciated as there are disparate viewpoints of these roles being more of a luxury rather than a necessity to the practice of laboratory medicine (1). The role of clinical consultation in the field of laboratory medicine can be particularly challenging to define as it rarely is accompanied by a formal consult note in the electronic medical record (EMR) and, thus, difficult to capture the value added to the healthcare team. Clinical consultation as it pertains to the diagnostic microbiology service often involves verbal or electronic guidance in the work up and interpretation of results to a broad range of healthcare professionals including laboratory scientists and physicians (2, 3). With expertise in all aspects of infectious diseases diagnostics, medical microbiologists are well equipped to offer clinical consultation.

To capture the breadth of clinical consultation and provide greater granularity to the value provided by medical microbiologists, we conducted an observational survey of consults received by the medical microbiology service at seven institutions and categorized them by impact.

## MATERIALS AND METHODS

### Study sites and service description

Study sites included the clinical microbiology laboratories at Children’s Hospital Los Angeles, Hospital of the University of Pennsylvania, Barnes–Jewish Hospital, Nationwide Children’s Hospital, Mount Sinai Health System NY, Deaconess Health System, and the University of Maryland Medical Center. Consultative service was defined as being performed by a medical microbiologist (with or without trainees) who engages in the consults with clinical colleagues. In addition to clinical consults, some sites also perform daily clinical microbiology laboratory rounds to generate laboratory-based consults from laboratory staff. An overview of the study sites and their contributions to consult collection can be found in Table 1.

**TABLE 1:** Overview of study sites

	Number of beds at main site	Number of test/year	Number of medical microbiologists	Trainees involved in service	Internal consults (total 231)	External consults (total 224)	Total consults (total 455)
Children’s Hospital Los Angeles	400	200,000	2	2 Fellows	51 (22.1)	43 (19.2)	94 (20.7)
Hospital of the University of Pennsylvania	1,200	400,000	2	2 Fellows, rotating residents	23 (10.0)	64 (28.6)	87 (19.1)
Barnes–Jewish Hospital	1,300	450,000	4	2 Fellows, rotating residents	119 (51.5)	45 (20.1)	164 (36.0)
Nationwide Children’s Hospital	700	350,000	3	None	2 (0.9)	15 (6.7)	17 (3.7)
Mount Sinai Health System	1,171	600,000	2	Rotating residents	2 (0.9)	26 (11.6)	28 (6.2)
University of Maryland Medical Center	800	220,000	3	1 Fellow, rotating residents	12 (5.2)	14 (6.3)	26 (5.7)
Deaconess Health System	750^*a*^	350,000	1	None	22 (9.5)	18 (8.0)	40 (8.8)

^*a*^ Total beds in three main hospitals. Parenthetical numbers indicate percentage of the total in the column.

#### Children’s Hospital Los Angeles (CHLA)

The Clinical Microbiology and Virology Laboratories represent subspecialties within the Department of Pathology and Laboratory Medicine. The clinical laboratories offer diagnostic services to both inpatient and outpatient. The microbiology and virology laboratories manage roughly 200,000 tests per year, with the majority of tests performed for the 400-bed quaternary-care free-standing pediatric Medical Center. Clinical microbiology service duties are shared across two medical microbiologists and CPEP clinical microbiology fellows. Each service team member has a dedicated pager number, and the bulk of clinical consultations are received through the formal paging system. Other methods of communication include secured text messaging, emails, phone calls, and in-person consults. The medical microbiologist on service and the fellows will also complete daily clinical microbiology laboratory rounds to assist medical laboratory scientists (MLSs) or medical laboratory technicians (MLTs) and troubleshoot any issues. All consults are reviewed and discussed on a weekly basis. On average, the service receives 50 consults per week.

#### Hospital of the University of Pennsylvania (HUP)

The Clinical Microbiology Laboratory represents a section within the Department of Pathology and Laboratory Medicine. The laboratory offers testing across all subspecialties of clinical microbiology. It is the main clinical microbiology laboratory for three hospitals, including the 1,000+ bed quaternary-care Hospital of the University of Pennsylvania. The laboratory manages roughly 400,000 tests per year. The clinical microbiology service consists of rotating pathology residents, CPEP clinical microbiology fellow(s), and two medical microbiologists. Beyond standard communications, the service receives consults via a dedicated clinical microbiology service secure chat group embedded in the electronic medical record and an “approval log” where orders requiring review and consultation are recorded by the laboratory and reviewed efficiently by a member of the service team. The medical microbiologist on service and the trainee team will also complete daily clinical microbiology laboratory rounds to assist MLSs or MLTs and troubleshoot any issues. On average, the service receives 75 consults per week.

#### Barnes–Jewish Hospital (BJH)

The clinical microbiology laboratory offers testing across all subspecialties of clinical microbiology. It is the main clinical microbiology laboratory for six hospitals, including the 1,300-bed Barnes–Jewish Hospital. The microbiology laboratory manages over 450,000 tests per year (this excludes most molecular infectious disease testing). The clinical microbiology service consists of rotating pathology residents, CPEP clinical microbiology fellow(s), and a medical microbiologist (four total) from Washington University School of Medicine. Beyond standard communications, the service receives consults via a dedicated clinical microbiology resident and fellow service phones. A subset of tests are routinely placed in an “approval log” where orders requiring review and consultation are recorded by the laboratory and reviewed efficiently by a member of the service team. The service team also completes daily clinical microbiology laboratory rounds to assist MLSs or MLTs and troubleshoot any issues. On average, the service receives 75 consults per week.

#### Nationwide Children’s Hospital

The laboratory offers testing across all subspecialties of clinical microbiology. It is the main clinical microbiology laboratory for the hospital including the 700-bed quaternary-care hospital in addition to 10 urgent care locations and an off-site emergency room. The clinical microbiology laboratory manages roughly 350,000 tests per year. The clinical microbiology service consists of three medical microbiologists that rotate responsibility. Beyond standard communications, the service receives communications by pager or email. On average, the service receives 15 external consults per week.

#### Mount Sinai Health System

The laboratory offers testing across all subspecialties of clinical microbiology. It is the main microbiology laboratory for eight hospitals in The Mount Sinai Health System, including the 1,171-bed quaternary-care Mount Sinai Hospital. The laboratory manages roughly 600,000 tests per year. The clinical microbiology service consists of rotating pathology residents, and two medical microbiologists. Beyond standard communications, the service receives consults via a dedicated email hashtag group and an on-call phone that is manned by rotating pathology residents during daytime business hours. On average, the service receives 50 consults per week.

#### University of Maryland Medical Center

The laboratory offers testing across all subspecialties of clinical microbiology and is the clinical microbiology laboratory for 14 hospitals across the University of Maryland Medical System. The laboratory manages roughly 220,000 tests per year, with the majority of tests performed for the 800-bed quaternary-care University of Maryland Medical Center. Clinical microbiology service duties are shared across three medical microbiologists, a CPEP clinical microbiology fellow, and rotating pathology residents. Beyond standard communications, consultations are received through a secure text messaging app to a dedicated clinical microbiology consult team account. On average, the service receives 75 consults per week.

#### Deaconess Health System

The laboratory offers testing across all subspecialties of clinical microbiology and has a select menu of molecular infectious disease testing. It serves as the centralized microbiology laboratory for five hospitals of the larger health system in Indiana and Kentucky, including 750 beds across three main sites. The laboratory also provides services for ambulatory locations within the health system and is a reference laboratory for inpatient nursing facilities, private provider offices, and non-Deaconess-affiliated entities in the region. The laboratory manages roughly 350,000 tests per year. Clinical microbiology service oversight is provided by one medical microbiologist. On average, the service receives 50 consults per week.

### Tracking consults

Study participants were asked to track and document consults received to the clinical microbiology service for a 1- to 2-week period in the Fall of 2022. Information recorded related to the consult included the mode of contact and by whom the consult was initiated, the member of the clinical microbiology service team that received the question, the patient setting, related laboratory section, a summary of the consult, recommendation and time spent in providing assistance, and if the medical microbiologist’s recommendation was accepted by the consulting individual. Consults were also tracked to determine whether the origin was internal (laboratory-based) or external (hospital-based). Internal was defined as consults coming from laboratory staff including MLS, MLTs, or laboratory medicine trainees, and external was defined as consults coming from any member of the healthcare team that is not from the laboratory. This would include physicians, pharmacists, medical students and infection control and preventionist.

### Assessing consult impact and opportunity

Consults were categorized by whether the recommendation directly or indirectly impacted patient care. Consults were further scrutinized for secondary outcomes, including if the interaction prompted an opportunity for diagnostic stewardship, education, cost reduction, or test guidance.

The following definitions were used for each to help guide assessment:

#### Direct impact

A recommendation that could change management for the present patient being discussed.

#### Indirect impact

A recommendation that could impact future patients but does not impact the care of a single patient.

#### Diagnostic stewardship

Reduction or more appropriate use of laboratory resources.

#### Education

Providing an explanation or justification of a process, discrepancy analysis, or result interpretation that could resolve confusion or improve care in the future.

#### Cost savings

Reasonable expectation that the recommendation eliminated additional expense inside or outside the laboratory (e.g. test or procedure avoidance).

#### Test guidance

Influencing test selection, optimal specimen collection, or result interpretation.

Consults were evaluated for these criteria by two blinded authors, one from the reporting institution and one from outside the reporting institution. A third review was conducted (K.G.R. or J.D.B) to adjudicate any discrepancies between the reviewers’ interpretations.

## RESULTS

Across the study period, a total of 455 consults were logged at all seven sites. The number of consults from each location varied widely, with the most being 164 and the fewest 18. Differences in the number of consults logged included the size of the institution and clinical microbiology laboratory, structure of daily operations and the service, and random variability in consult frequency across the small study window. Table 2 summarizes the consults received.

**TABLE 2:** Description of consults received^*a*^

		Internal consults (total 231)	External consults (total 224)	Total consults (total 455)
How was consult initiated?	Clinical microbiology laboratory rounds	108 (46.8)	4 (1.8)	112 (24.7)
	Phone call	40 (17.3)	62 (28.3)	102 (22.5)
	Text	n/a	69 (31.5)	69 (15.2)
	Page	17 (6.9)	21 (9.6)	37 (8.1)
	Email	17 (7.4)	42 (19.2)	59 (13.0)
	Face-to-face	50 (21.6)	5 (2.3)	55 (12.1)
	Approval log	n/a	21 (9.6)	21 (4.6)
Who sought consult?	MLS/MLT	214 (92.6)	0 (0)	214 (47.1)
	Lab management	13 (5.6)	0 (0)	13 (2.9)
	Physician (ID)	0 (0)	118 (53.9)	118 (26.0)
	Physician (non-ID)	0 (0)	62 (28.3)	62 (13.7)
	Nurse practitioner/physician assistant/advanced practice provider	0 (0)	11 (5.0)	11 (2.4)
	Nursing	0 (0)	6 (2.7)	6 (1.3)
	CPEP fellow	1 (0.4)	0 (0)	1 (0.2)
	Infection prevention and control	0 (0)	15 (6.8)	15 (3.3)
	Antimicrobial stewardship pharmacist	0 (0)	4 (1.8)	4 (0.9)
	Client services	3 (1.3)	0 (0)	3 (0.7)
	Pharmacist	0 (0)	5 (2.3)	5 (1.1)
	Unknown	0 (0)	3 (1.4)	3 (0.7)
Who received consult?	Lab director	127 (55.0)	107 (48.9)	234 (51.5)
	Fellow	75 (32.4)	97 (44.3)	172 (37.8)
	Resident	29 (12.6)	20 (9.1)	49 (10.8)
Patient environment	Inpatient	113 (48.9)	180 (82.2)	293 (64.4)
	Outpatient	65 (28.1)	35 (16.0)	100 (22.0)
	Emergency department	23 (10.0)	4 (1.8)	27 (5.9)
	Satellite hospital	21 (9.1)	3 (1.4)	24 (5.3)
	Unknown	9 (3.9)	2 (0.9)	11 (2.4)
Lab section	Bacteriology	177 (76.6)	88 (40.2)	265 (55.9)
	Mycology	10 (4.3)	32 (14.6)	42 (8.9)
	Parasitology	3 (1.3)	6 (2.7)	9 (1.9)
	Molecular: high complexity	10 (4.3)	50 (22.8)	60 (12.7)
	Molecular: sample-to-answer	16 (6.9)	21 (9.6)	37 (7.8)
	Serology	7 (3.0)	12 (5.5)	19 (4.0)
	Virology	2 (0.9)	2 (0.9)	4 (0.8)
	Mycobacteriology	4 (1.7)	6 (2.7)	10 (2.1)
	Infectious disease pathology	0 (0)	6 (2.7)	6 (1.3)
	Not specific	2 (0.9)	1 (0.5)	3 (0.6)
Consult type	Culture workup	136 (58.9)	37 (16.9)	173 (38.0)
	Specimen acceptability	18 (7.8)	7 (3.2)	25 (5.5)
	Test approval	22 (9.5)	56 (25.5)	78 (17.2)
	Test ordering guidance	1 (0.4)	24 (11.0)	25 (5.5)
	Result interpretation	11 (4.8)	40 (18.3)	51 (11.2)
	Send out testing	9 (3.9)	18 (8.2)	27 (5.9)
	Antimicrobial guidance	18 (7.8)	39 (17.8)	57 (12.5)
	Lab management	8 (3.5)	0 (0)	8 (1.8)
	Standard operating procedure	2 (0.9)	0 (0)	2 (0.4)
	Result estimated time of arrival	0 (0)	2 (0.9)	2 (0.4)
	Critical result notification	6 (2.6)	0 (0)	6 (1.3)
	EMR related	0 (0)	1 (0.5)	1 (0.2)
Time spent on consult	Less than 5 min	124 (53.7)	85 (39.4)	209 (44.8)
	5–15 min	69 (29.9)	85 (39.4)	154 (33.0)
	15–30 min	27 (11.7)	32 (14.8)	59 (12.7)
	30–60 min	6 (2.6)	13 (6.0)	19 (4.1)
	Greater than 1 h	1 (0.4)	2 (0.9)	3 (0.6)
	Unknown	4 (1.7)	7 (3.2)	11 (2.4)
Recommendation accepted?	Yes	178 (77.1)	181 (82.6)	359 (78.2)
	No	4 (1.7)	2 (0.9)	6 (1.3)
	NA	45 (19.5)	20 (9.1)	65 (14.2)
	Unknown	4 (1.7)	21 (9.6)	25 (5.4)

^*a*^ Parenthetical numbers indicate percentage of the total in the column.

The origin of microbiology consults was split evenly between internal (231) and external (224) to the laboratory. In internal to the laboratory, the majority (93%) of questions originated from MLSs or MLTs via many modes of communication, but nearly half occurred during clinical microbiology laboratory rounds. Overwhelmingly, the majority (77%) of internal consults were related to the analytical phase of testing, including clarification of the standard operating procedure (SOP), guidance with challenging specimen processing or culture workup, and approval for special test requests. These highlight two major areas of value provided by medical microbiologist leadership: diagnostic work-up guided by clinical context and diagnostic and/or antimicrobial stewardship. An example seen commonly in the survey included appropriate organism reporting on complex cultures when multiple isolates or uncommon pathogens were encountered. This is an important opportunity for the medical microbiologist to impact proper patient management, bringing the treating team’s attention to important pathogens while limiting the reporting and additional workup of insignificant findings. Outcomes of exercising such expertise include conservation of laboratory resources, avoidance of unnecessary treatment, and appropriately targeting therapy.

External consults were most commonly initiated by physicians (82.2%), with two-thirds coming from infectious diseases physicians (Table 2). Unsurprisingly, these consults centered around pre-analytic components of testing, including discussions about best test selection, approval for testing, and selection of appropriate antimicrobial susceptibility testing, or the post-analytical phase of testing, including result interpretation and discussion on the value of additional diagnostic options or culture workup. Across the board, >75% of consults took <15 min to complete and with just over half of all the internal questions being answered in <5 min. Interestingly, while external consults were also largely completed within the 15-min timeframe, they were split evenly in the 1- to 5- and 5- to 15-min group, suggesting some increased complexity for requests or time needed for conversation. Nevertheless, these data show that medical microbiologists are devoting significant portions of their day to consultation, while fulfilling all the other aforementioned roles. Regardless of the consult being internal or external, the recommendation of the medical microbiologist was almost universally followed (98.9%).

As summarized in Table 3, the majority (74.9%) of questions fielded by medical microbiologists were determined to have direct patient impact. For example, multi-drug-resistant pathogens present significant challenges for patient management. Medical microbiologists can assist in determining the likely antimicrobial resistance mechanism(s), interpretation of susceptibility testing results, and guide testing of potentially useful extended spectrum antimicrobials. Therefore, medical microbiologists can contribute to targeted management and speed to the most appropriate therapy, both important roles to assist successful antimicrobial stewardship teams (4). These data highlight the “team-of-teams” aspect of healthcare delivery and the integral role played by medical microbiologists in collaborative patient management. Both internal and external consults presented opportunities for impact beyond the individual patient. However, external consults consistently provided more opportunity for diagnostic stewardship (33.9% external vs 22.9% internal) and education (52.2% external vs 29.0% internal). Internal consults created more opportunity for cost reduction (31.2% internal vs 22.3% external), driven largely by reduction of culture workup or additional testing. Test guidance opportunities were effectively equal between the groups (45.5% internal vs 42.9% external) (Table 3). Examples of consults that were received within the study timeframe are summarized in Table 4.

**TABLE 3:** Impact of clinical microbiologist consultation

	Patient impact		Diagnostic stewardship opportunity		Education opportunity		Cost reduction opportunity		Test guidance opportunity	
	Direct (%)	Indirect (%)	Yes (%)	No (%)	Yes (%)	No (%)	Yes (%)	No (%)	Yes (%)	No (%)
External consults	165 (73.7)	59 (26.3)	76 (33.9)	148 (66.1)	117 (52.2)	107 (47.8)	50 (22.3)	174 (77.7)	96 (42.9)	128 (57.1)
Internal consults	176 (76.2)	55 (23.8)	53 (22.9)	178 (77.1)	67 (29.0)	164 (71.0)	72 (31.2)	159 (68.8)	105 (45.5)	126 (54.5)
Total consults	341 (74.9)	114 (25.1)	129 (28.4)	326 (71.6)	184 (40.4)	271 (59.6)	122 (26.8)	333 (73.2)	201 (44.2)	254 (55.8)

**TABLE 4:** Snapshot of consults received by the medical microbiologist

Consult type	Question from	Patient setting	Laboratory specialty	Consult category		Action/ recommendation	Time spent	Recommendation accepted?	Comments
Face-to-face	Physician: Non-ID	Inpatient	ID pathology	Result interpretation	Consult on histopathology slides; tissue blocks with budding yeast	Recommended reporting broad-based budding consistent with *Blastomyces* species; recommend correlation with microbiology cultures	5–15 min	Yes	Consult provided assistance in determining the likely infectious organism
Phone call	Physician: non-ID	Outpatient	Molecular: high complexity	Test ordering guidance	Request for add on herpes simplex virus (HSV) PCR and Venereal Disease Research Laboratory for amniotic fluid	Testing options were discussed with clinician. Recommended screening mother first, as this is very rare and would either be off-label testing with possibility of reference lab performing. Ultimately HSV and syphilis screening tests ordered on mother	5–15 min	Yes	Consult resulted in the ordering of appropriate tests for diagnosis as well as presented an opportunity for laboratory stewardship
Phone call	Physician: ID	Outpatient	Serology	Result interpretation	Reactive surface antigen, surface antibody negative, as well as core IgM, core IgG/IgM negative, DNA negative, e Ag/Ab both negative	Interpret as likely false positive; recommend retesting in approximately 4–6 weeks	15–30 min	Yes	Consult resulted in the correct interpretation of testing results, with appropriate follow up testing recommendations
Email	Physician: ID	Inpatient	Molecular: high complexity	Result interpretation	Broad-range PCR detected six bacteria in a sample of jaw tissue. Consult to aid in the interpretation of molecular result	After review and comparison with culture, which was negative for any microbial growth, provided interpretation to clinician that findings were highly likely insignificant and represented normal flora	5–15 min	Yes	Consult resulted in avoiding unnecessary treatment for multiple organisms detected by PCR only
Email	Physician: non-ID	Inpatient	Molecular: sample-to-answer	Result interpretation	Physician has patient with multiple respiratory viral panels that were positive for both influenza A and B. Wanted to know if it was possibly the vaccine they received that could cause that result	Discussed interpretation of the test and informed them that live attenuated vaccines can be detected by the panel. Given that patient received nasal vaccine, it is a possibility	30–60 min	Yes	Consult provided further information about the test and test result that was helpful in patient management
Email	Physician: non-ID	Inpatient	Molecular: sample-to-answer	Test approval	Physician reached out to request repeat multiple respiratory viral panels on a patient that had one performed 16 h prior. Requesting on behalf of the transplant team who "wanted to make sure that the previous panel did not miss anything" before they change his immunosuppression regimen to manage new manifestation of graft-versus-host disease	Reject. Explained that less than 24 h is not an appropriate amount of time to retest. Explained that it is a very sensitive test so not likely to miss anything in 24 h from properly collected specimen. Communicated that we could revisit again in a few days if the team still feels strongly	15–30 min	Yes	Consult resulted in avoiding unnecessary testing and a cost-saving measure for the laboratory and patient
Text	Physician: ID	Inpatient	Bacteriology culture	Antimicrobial guidance	Physician requested explanation as to why antimicrobial susceptibility testing (AST) results did not have MIC listed for cefazolin and just the interpretation for a methicillin-susceptible *Staphylococcus aureus* from blood culture	Explained that cefazolin can be inferred from oxacillin per the Clinical and Laboratory Standards Institute (CLSI) and oxacillin was susceptible with MIC listed. CLSI document offered guidance on the situation	5–15 min	Yes	Consult resulted in the appropriate interpretation of AST results for the purpose of patient treatment.
Phone call	Physician: non-ID	Outpatient	Molecular: high complexity	Result interpretation	Requesting explanation of how path showed condyloma but human papillomavirus (HPV) assay on tissue was negative	Discussed that while the PCR assay covers a wide number of HPV strains, it is not all inclusive	5–15 min	Yes	Consult resulted in the correct interpretation of high complexity molecular testing in relation to other test results
Text	Physician: ID	Inpatient	Molecular: high complexity	Test approval	Karius test requested for cancer center patient with potential aortic graft infection. Unable to biopsy, and patient presents with fever of unknown origin that appear after each chemo treatment. Patient now qualifies as septic	Denied the request. Patient is septic and had blood cultures that were not even 24 h old. Further, the cancer team was considering possible cancer spread rather than infection. Blood cultures grew yeast and bacteria	Greater than 1 h	Yes; ID team not happy with decision at first, accepted decision once cultures grew	Consult resulted in an opportunity for diagnostic stewardship, prevented sending out for unnecessary testing that would have given the same results as the culture and prevented spending of high-dollar test
Face-to-face	Lab: MLT	Inpatient	Bacteriology culture	Specimen acceptability	Catheter tip submitted for culture. Do we process?	Denied the request. We no longer accept catheter tip culture as it is an inferior specimen than corresponding body fluid [blood, peritoneal fluid, cerebrospinal fluid (CSF)]. Patient was already growing *Staphylococcus epidermidis* from multiple blood cultures	Less than 5 min	Yes; test canceled without objection	Consult prevented testing of inferior specimen that would only serve to introduce potential contamination into patient diagnostic workup
Page	Lab: MLS	Inpatient	Bacteriology culture	Culture Workup	MLS reached out because they had identified fungal elements in Gram stain of a positive blood culture, but when they made a critical call to the doctor, they said the patient had GNRs at outside hospital. MLS was unsure of how to proceed	Reviewed Gram stain images and confirmed Gram-negative rods (GNRs). Reached out to lab director to consult and confirm that the images correlated with GNR. Recommended setting up rapid identification testing to confirm. Called the doctor and discussed repeat blood cultures and let them know it is most likely GNR but we are confirming. Reported GNR to patient record. Gram stain appeared to be filamentous GNRs or GNRs with antibiotic effect so called the provider to let them know to prevent treatment with antifungals. blood culture identification panel identified the organism as *Salmonella* species	30–60 min	Yes; physician appreciated the call and was relieved to know that since it was GNR we would be performing susceptibility testing	Consult resulted in avoiding potential use of antifungals on a patient being treated for GNR bacteremia. Presented opportunity to discuss our workup with provider and provide coaching/mentorship to the young MLS that made the initial error. Lab director implemented criteria to consult if suspected fungus seen on Gram stain from sterile body sites
Text	ASP	Inpatient	Bacteriology culture	Test ordering guidance	A Doctor of Pharmacy was questioning the need for blood cultures ordered by provider to "document clearance" from Gram-negative bacteremia	Advised no blood cultures needed if clinically improved. Cancel blood cultures. Patient clinically responded to therapy, afebrile, stable, normal white blood cells, no indication for new or persistent infection	5–15 min	Yes	Consult resulted in both an opportunity for laboratory stewardship and for education
Plate rounds	Lab: admin/management	Outpatient	Bacteriology culture	Antimicrobial guidance	Review of sterile site infection found therapeutic error. Patient growing *Candida glabrata* from pelvic fluid and on fluconazole. Contacted ID to change to echinocandin	Request to change therapy. Fluconazole not the preferred treatment for serious *C. glabrata* infections	15–30 min	No; going to increase dose and see how patient does	Consult resulted in the lab director implementing criteria to consult if suspected fungus seen on Gram stain from sterile body sites
Phone call	Physician: ID	Inpatient	Parasitology	Test ordering guidance	Woman with HIV and watery diarrhea. Recommendation for further testing and review of surgical pathology (SP) with pathology including Whipple’s	Reviewed SP, suggested second O + P and consider molecular parasite panel	30–60 min	Unclear	Consult resulted in recommendations for follow up testing that may aid in diagnosis
Face-to-face	Lab: MLS	Outpatient	Bacteriology culture	Test approval	Cefpodoxime for *Enterococcus faecalis* and *Pseudomonas aeruginosa*	Not approved. Discussed with provider that these are inherently resistant	5–15 min	Yes	Consult resulted in both laboratory stewardship as well as education on treatment for these organisms
Phone call	Lab: MLS	Outpatient	Serology	Test approval	*Aspergillus fumigatus* IgG serology	Patient has positive skin test, elevated serum IgE, negative *Aspergillus* IgE, which does not meet testing criteria. Called provider who agreed to cancel test	Less than 5 min	Yes	Consult resulted in both laboratory stewardship and correct test result interpretation prior to follow up testing
Text	Physician: non-ID	Inpatient	ID pathology	Result interpretation	Pathology stain of bronchoalveolar lavage showed fungal forms; pathologist asking for help interpreting what type of fungi it could be	Fungal forms appeared aseptate, which correlated to the growth in bacterial and fungal cultures	Less than 5 min	Yes	Consult helped pathologist finish report and correlate findings in the pathological smears to those found in culture, resulting in clear picture for the ID team for treatment of patient with fungal abscess
Text	NPs	Inpatient	Molecular: high complexity	Test ordering guidance	Patient suspected of leptospirosis, unsure of what the best testing method is	Send for molecular and serology at the Centers for Disease Control. Molecular is gold standard for identification of leptospiral infections	15–30 min	Yes	Consult resulted in the appropriate tests selected for diagnosis
Phone call	RNs/other nurses	Outpatient	Bacteriology culture	Antimicrobial guidance	Nurse wants to know if cephalexin can be used to treat *E. faecalis* isolated from urine	Communicated that cephalosporins are not tested for *Enterococcus*. Suggested ampicillin and if needed to consult ID	5–15 min	Yes	Consult prevented possible improper use of antibiotic that would not be clinically effective

## DISCUSSION

To our knowledge, this study is the first to provide detailed evaluation of the consultative value provided by medical microbiologists. Medical centers and health systems recognize the criticality of qualifications in subspecialties of medicine that wholly benefits patients, and subspecialty training within pathology and laboratory medicine is no exception. The average weekly consults reported by the seven institutions and the complexity of consults received within the short timeframe measured highlight the need for doctoral-level medical directors with subspecialty training and board certification in medical microbiology. The analysis demonstrates that no matter the setting, when available, medical microbiologists are often utilized and respected members of the healthcare team. Colleagues nearly universally accept their recommendations, and their expertise is sought in all phases of the testing lifecycle.

Approximately 50% of all consults were from healthcare team members outside of the laboratory setting. Despite the ID subspecialty being more well versed in infectious diseases diagnostics compared to other subspecialities, the majority of physician consult requests were from our ID colleagues. We suspect this is related, at least in part, to existing relationships and familiarity of ID physicians with medical microbiologists through interdisciplinary committees (antimicrobial stewardship, infection prevention, etc.), co-attendance at ID rounds and microbiology plate rounds and overlapping interests. However, their willingness to seek advice confirms that even clinicians most well versed in pathogen testing depend on their medical microbiology colleagues for guidance. Further, as the ID service often includes highly complexed cases, they often act as the liaison between the other care teams and microbiology. As the majority of microbiology tests and results are ordered and reported to non-ID colleagues, this highlights a vast potential for disseminating medical microbiology consultations to other subspecialties that would thoroughly benefit from guidance in microbiology test selection and result interpretation directly from the medical microbiologist.

Internal to the laboratories, the medical microbiologist provides invaluable guidance on testing processes to laboratory staff. While internal questions may not meet the traditional definition of a “consult,” capturing these interactions and codifying their benefits similar to external consults is relevant to the overall understanding of a medical microbiologist’s impact as a source of expertise. Medical microbiologists not only develop the protocols and training activities used by MLSs but are also involved in creating educational materials on testing principles, new assays, and emerging pathogens that provide important knowledge for the laboratory team. The critical shortage of MLSs is well documented, only exasperated by the challenges of the coronavirus disease 2019 pandemic (5). As the next generation of laboratory staff gains experience, expertise of the medical microbiologist is even more critical as they are able to maintain the highest level of quality results through close consultation with MLSs in the laboratory. Anecdotally, medical microbiologists involved in this study report an increase in the consult volume from the laboratory staff and greater need for involvement in daily operations and culture workup as a more novice workforce acclimates to the complex procedures of diagnostic microbiology. This highlights the importance of a medical microbiologist’s presence in the laboratory and how beneficial interfacing with the technologists can be in optimal diagnostic testing.

Whether expected as part of clinical oversight or formalized as a condition of faculty appointment, post-graduate education can be a major responsibility for a laboratory medical director. While consults are not traditionally captured in educational impact, considered instead to be a component of clinical service, our data show that ample opportunity for education across the health system exists in these interactions. Medical microbiologists taking the time to explain test performance, testing utility, or rationale behind appropriate antimicrobial susceptibility testing may promote more efficient and improved patient management at the next opportunity. In addition, these consults provide an interprofessional educational opportunity for medical microbiologists to elevate resident and fellow training across all specialties. Medical microbiologists can use consults as a springboard to in-depth discussion on particular topics or to help trainees gain experience in providing effective recommendations and hone their skills interacting across diverse clinical services.

Test complexity has increased in the clinical microbiology laboratory, with a boom in new technologies introduced to the field in the last decade. These include, but are not limited to, matrix-assisted laser desorption ionization–time-of-flight mass spectrometry and sequencing for isolate identification, an expansive menu of molecular assays for pathogen detection, syndromic testing and antimicrobial resistance prediction, new antimicrobials and accompanying susceptibility testing methods, and sequencing-based diagnostic tests. The number of consults related to these new methods highlight the challenge in keeping up with diagnostic advancement and creates opportunity for medical microbiologists to guide clinicians to the right test(s) at the right time and assist in result interpretation. Additionally, some of the newer applications, namely, sequencing-based pathogen detection tests, can cost thousands of dollars per test, with limited clinical utility outside specific patient populations or clinical syndromes. The medical microbiologist serves an important role in diagnostic stewardship of these tests, facilitating and recommending use when warranted, but protecting the health systems and patients from exorbitant costs in cases when little is to be gained (6). Moreover, when these clinical tests are ordered, medical microbiologists are primed to serve as the subject matter expert to guide the standardized reporting of results and aid in the interpretation of the evermore complex results (7).

A highly speculative editorial published in an Intensive Care Medicine journal argues that artificial intelligence and emerging automated technologies will rapidly reduce the need for medical microbiologists (8). One might argue that the same logic may be inferred to any other subspecialties of medicine. The simplicity of the statements made in the article underscores the lack of comprehension of the complexity of the field of laboratory medicine as a whole. As great as new technologies are, they are not infallible, and the role of artificial intelligence is still yet to be determined, especially with the ever-changing nature of microbes. A unique skill of medical microbiologists is the ability to review and interpret Gram stain and histopathology slides, and as demonstrated in this study, it is not uncommon for anatomic pathologists to consult their medical microbiology colleagues on suspected infectious cases. Our study provides evidence that medical microbiologists will continue to provide high-value expertise in the next frontier of diagnostic microbiology. Greater than half of the external consults pertained to the appropriate selection, performance, and interpretation of diagnostics, frequently pertaining to high-complexity molecular assays. Our patient-facing colleagues are leaning on their local microbiology expert for advice. We expect that, as assay complexity increases (e.g., greater adoption of next-generation sequencing-based assays, training and maintenance of machine-learning algorithms for slide reading, or use of whole-genome sequencing-based antimicrobial resistance prediction), the role for the medical microbiologist in all phases of testing will become increasingly vital (9). Beyond the direct testing aspects and clinical consultation, the expertise of the medical microbiologist will be paramount in guiding the selection of the most impactful advancements in the context of individual laboratory and hospital needs. For instance, as budgets are tightened, medical microbiologists will increasingly practice laboratory stewardship to assess clinical need and relevance of new technologies to maximize impact while being financially judicious. As recently described by Culbreath et al., adoption of automation in the clinical microbiology laboratory does not reduce the need for expertise, but instead allows for acceptance of increased volumes of specimens without the need for a corresponding increase in staffing (10). This allows laboratory staff to spend more time evaluating complex cultures and expands the need for consultations by medical microbiologists.

Limitations of our study include the number of sites included in the survey, a bias toward academic medical centers, and the limited duration of data collection. First, as our study was very heavily influenced by academic medical centers where a medical microbiologist is often employed, these data may not fully represent smaller laboratories, community hospitals, or those with a more general pathology director without medical microbiology training and board certification overseeing the microbiology laboratory. Inclusion of more healthcare settings in future studies will be important to measure the impact of medical microbiology consults as a whole. Second, sites were given freedom to record consults over a 1- to 2-week period without additional limitations. The amount of data collected may itself be variable week to week, with increased or decreased volumes observed during collection in comparison to average. As the study was only performed over a 2-week period, it very likely did not capture the full scope and complexity of consults. For instance, one of the sites involved only documented 26 consults during the 2-week study period; however, they independently documented nearly 100 consults in the following 2-week period. Site-specific volume, coupled with variation across the short study window, resulted in a wide range of contributions from each site, with three locations (BJH, CHLA, HUP) making up 76% of the data. To eliminate concern that data imbalance was biasing results based on these three sites and not reflecting the study sites as a whole, data were reanalyzed with the top contributors removed. Overall conclusions remained the same, with frequency of consult characteristics, patient impact, and secondary opportunities shifting insignificantly from the full data set (data not shown). This confirms that while some sampling bias may exist in our study’s small snapshot, high-level trends in medical microbiologist consultation are shared across sites. In this study, it was noted that not all sites perform daily clinical microbiology laboratory rounds, and although they may receive questions from the laboratory, not all questions were logged as a formal consult and may have decreased overall volume for some sites.

Medical microbiologists perform a variety of integral roles, not the least of which is clinical consultation. Our study is the first to collect, categorize, and assess the value of these interactions. Our data show that the expertise of the medical microbiologist is sought by individuals across the healthcare team, both inside and outside the laboratory. Their advice is highly regarded, and recommendations often result in direct patient impact. However, there is ample opportunity remaining that will allow the medical microbiologist to provide additional value as a part of the clinical team. As noted in this study, the mode of communication lacked standardization ranging from face-to-face communication to an informal text message. One important initiative to prioritize is the standardization of consult communication within the EMR, such as the composition of consult notes and the integration of secure messaging that contacts the consult team directly. These activities mirror those of our clinical colleagues and may lead to further entrenchment as part of a clinical team, which has been shown to generate high levels of physician satisfaction (11). As health systems expand to include remote sites without a dedicated in-house medical microbiologist at all locations, a more formalized process for consultation and EMR integration could allow expanded opportunity to share expertise. Tools to document recommendations in the EMR, e.g., Epic iVent, are already in use by pharmacists to document antimicrobial stewardship recommendations (12). Adoption of a similar tool could facilitate a future where consultation of a medical microbiologist is more widely available to those in need of assistance.
